# The stereoisomeric *Bacillus subtilis* HN09 metabolite 3,4-dihydroxy-3-methyl-2-pentanone induces disease resistance in *Arabidopsis* via different signalling pathways

**DOI:** 10.1186/s12870-019-1985-6

**Published:** 2019-09-05

**Authors:** Niu Liu, Xiao Luo, Yongqing Tian, Duo Lai, Longlai Zhang, Fei Lin, Hanhong Xu

**Affiliations:** 1 State Key Laboratory for Conservation and Utilization of Subtropical Agro-Bioresources/Key Laboratory of Natural Pesticide and Chemical Biology, Ministry of Education, South China Agricultural University, Guangzhou, 510642 China; 2 Guangdong Province Key Laboratory of Microbial Signals and Disease Control, South China Agricultural University, Guangzhou, 510642 China; 3 Key Laboratory of South Subtropical Fruit Biology and Genetic Resource Utilization, Ministry of Agriculture, Guangdong Academy of Agricultural Sciences, Guangzhou, 510640 China

**Keywords:** PGPR, ISR, Metabolite, 3,4-dihydroxy-3-methyl-2-pentanone, Stereoisomer

## Abstract

**Background:**

Plant immune responses can be induced by plant growth-promoting rhizobacteria (PGPRs), but the exact compounds that induce resistance are poorly understood. Here, we identified the novel natural elicitor 3,4-dihydroxy-3-methyl-2-pentanone from the PGPR *Bacillus subtilis* HN09, which dominates HN09-induced systemic resistance (ISR).

**Results:**

The HN09 strain, as a rhizobacterium that promotes plant growth, can induce systemic resistance of *Arabidopsis thaliana* plants against *Pseudomonas syringae* pv. *tomato* DC3000, and the underlying role of its metabolite 3,4-dihydroxy-3-methyl-2-pentanone in this induced resistance mechanism was explored in this study. The stereoisomers of 3,4-dihydroxy-3-methyl-2-pentanone exhibited differential bioactivity of resistance induction in *A. thaliana*. B16, a 1:1 mixture of the threo-isomers (*3R,4S*) and (*3S,4R*), was significantly superior to B17, a similar mixture of the erythro-isomers (*3R,4R*) and (*3S,4S*). Moreover, B16 induced more expeditious and stronger callose deposition than B17 when challenged with the pathogen DC3000. RT-qPCR and RNA-seq results showed that B16 and B17 induced systemic resistance via JA/ET and SA signalling pathways. B16 and B17 activated different but overlapping signalling pathways, and these compounds have the same chemical structure but subtle differences in stereo configuration.

**Conclusions:**

Our results indicate that 3,4-dihydroxy-3-methyl-2-pentanone is an excellent immune elicitor in plants. This compound is of great importance to the systemic resistance induced by HN09. Its threo-isomers (*3R,4S*) and (*3S,4R*) are much better than erythro-isomers (*3R,4R*) and (*3S,4S*). This process involves SA and JA/ET signalling pathways.

**Electronic supplementary material:**

The online version of this article (10.1186/s12870-019-1985-6) contains supplementary material, which is available to authorized users.

## Background

Plant growth-promoting rhizobacteria (PGPRs) that colonize host roots can improve disease resistance and promote plant growth [1–3]. Most PGPRs generate a series of secondary metabolites [4] as the elicitors of plant defense response, thus directly or indirectly triggering induced systemic resistance (ISR). To date, many elicitors have been characterized. M-cresol and methyl benzoate (MeBA), as active volatile compounds from *Ampelomyces sp.* and *Cladosporium sp.*, respectively, can produce ISR against pathogens [5]. The volatile organic compounds (VOCs) 2,3-butanediol and 3-methyl-1-butanol identified from *B. subtilis* GB03 and *B. amyloliquefaciens* IN937a confer ISR against the bacterial pathogen *Erwinia carotovora* subsp. Carotovora [1, 6]. Exopolysaccharides, 2,3-butandiol, bacilysin, difficidin, macrolactin, bacillaene, surfactin, bacillomycin D and fengycin, which are *B. amyloliquefaciens* SQR9 metabolites can improve systemic resistance against *P. syringae* pv. *tomato* DC3000 and *Botrytis cinerea* [7].

After elicitors are recognized, ISR is triggered in plants by activation of jasmonic acid (JA), ethylene (ET) and salicylic acid (SA) signalling [8], which are interconnected by complex signalling networks and crosstalk [9]. The molecular basis of this type of ISR has been widely described. From *A. thaliana* mutants impaired in JA or ET signalling, it was found that JA and ET can regulate rhizobacterium-mediated ISR [10]. Proteomic analysis indicated that bacterial volatiles produced by *B. subtilis* GB03 significantly induced the upregulation of ET biosynthesis enzymes [11]. *B. amyloliquefaciens* NC6 can produce the PeBA1 protein, which induces systemic resistance against pathogens, including fungal pathogen *B. cinerea* and tobacco mosaic virus (TMV). After treatment with PeBA1, the SA-responsive *PR1a*, *PR1b*, *PR5*, and *PAL*, as well as the JA-responsive *PDF1.2* and *COI1*, were upregulated.

The chiral configuration of elicitors significantly affects their biological activity. GB03-synthesized (2R,3R)-butanediol triggered ISR against *Erwinia carotovora* ssp*. carotovora* SCC1 in *Nicotiana tabacum*, but another stereoisomer, (2S,3S)-butanediol, did not have the same effect [1, 12]. Similarly, herbivore elicitor volicitin has obvious structural specificity of insect-derived elicitors. The compound has active L-isomer, but inactive D-isomer [13]. The existence of stereoisomer specificity has been demonstrated in PGPR metabolites, but reports on the comparison of specific ISR signaling pathways activated by chiral compounds are limited, partly due to the technical difficulty associated with separating stereoisomers. Although chiral compounds are structurally similar, they are actually two different substances and often have distinct functions. The separation of chiral compounds and comparison of the resistance-related signalling pathways activated by these compounds are important. Such studies understand the plant immune responses mechanically and allow identification of novel chemical inducers through rational design.

*B. subtilis* HN09 induces resistance to many plant diseases depending on the secondary metabolites. The (*3R,4S*) and (*3S,4R*) 3,4-dihydroxy-3-methyl-2-pentanone 1:1 racemate B16 and (*3R,4R*) and (*3S,4S*) 3,4-dihydroxy-3-methyl-2-pentanone 1:1 racemate B17 were isolated from HN09 culture filtrate and were verified to be involved in the HN09-induced systemic resistance to *P. syringae* pv. *tomato* DC3000 in *A. thaliana*. Our data showed a significant difference between the abilities of B16 and another pair of stereoisomers, B17, to elicit defence responses at the plant, cellular and molecular levels.

## Results

### HN09 metabolites induce resistance in *A. thaliana*

**Fig. 1:**
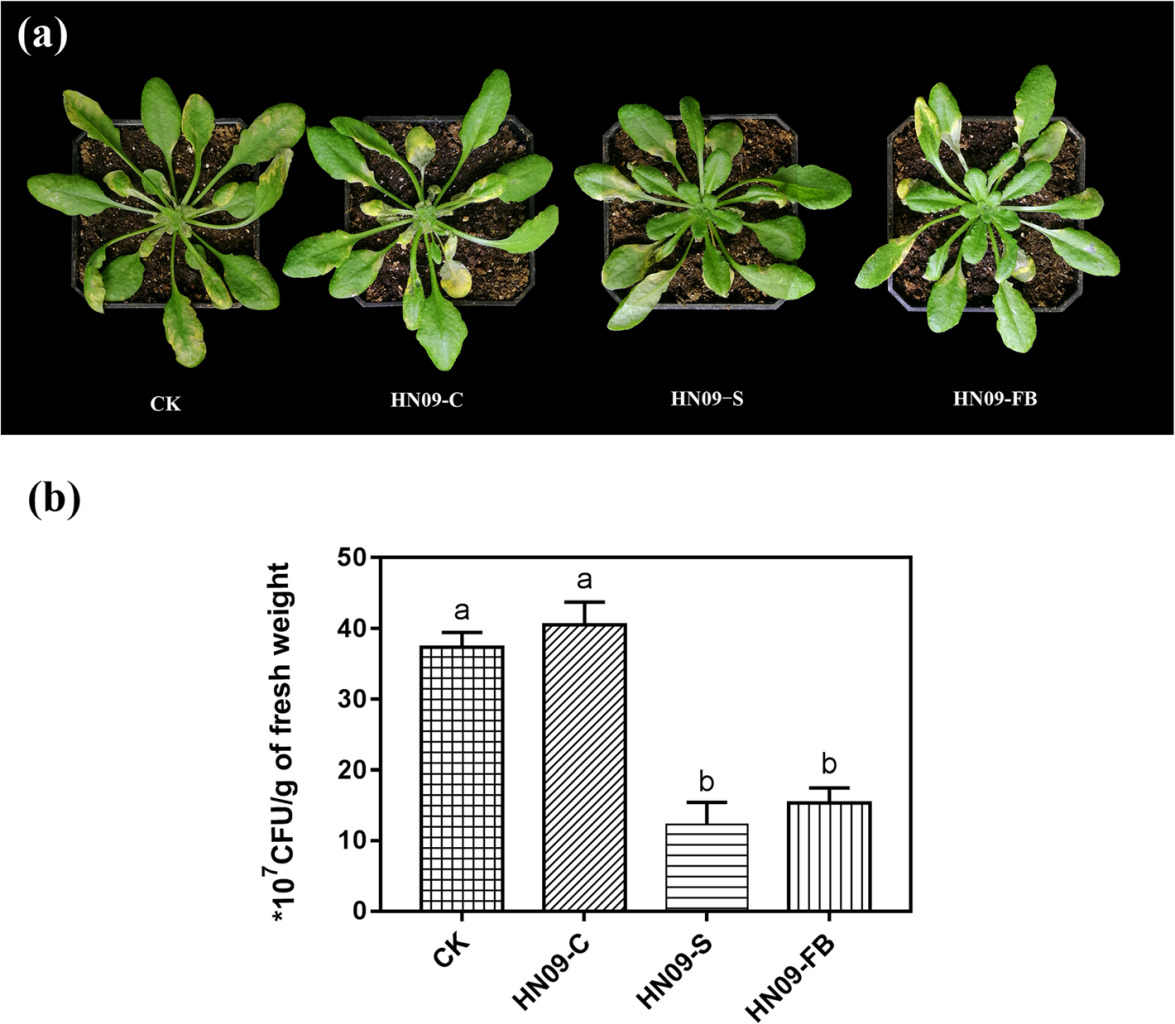
HN09 metabolites induce resistance in *A. thaliana*. Induction of systemic resistance to *Pseudomonas syringae pv. tomato* DC3000 in *A. thaliana* Col-0 plants treated with HN09 and its fermentation broth. *A. thaliana* Col-0 plants were treated with HN09 bacteria cells (HN09-C), HN09 fermentation broth at OD_600_ = 0.1 (HN09-FB), supernatant of HN09 fermentation broth (HN09-S) and 10 mM MgSO_4_ (CK). Five days later, leaves were sprayed with a cell suspension of DC3000 at OD_600_ = 0.02. (**a**) Representative plants of HN09-C, HN09-S and HN09-FB were photographed 5 days post inoculation (dpi). (**b**) Colony density of DC3000 in the leaves of *A. thaliana* plants of different treatment groups. Values are the average CFU per gram of leaf; each treatment had 12 plants, and 3 leaves per plant were obtained for quantification of DC3000 density (least significant difference test; *P* < 0.05). All experiments were conducted twice with similar results

The metabolites of PGPRs play important roles in ISR. PGPR *B. amyloliquefaciens* SQR9 can induce systemic resistance against *P. syringae* pv. *tomato* DC3000 and *Botrytis cinerea.* However, the mutants of SQR9 strain, which can weaken the induced plant resistance to phytopathogens, were deficient in the production of extracellular compounds, including exopolysaccharides, 2,3-butandiol, bacilysin, difficidin, macrolactin, bacillaene, surfactin, bacillomycin D and fengycin [7]. *B. subtilis* HN09 strongly enhanced systemic resistance to *Magnaporthe oryzae* and *Fusarium oxysporum* f. sp. *radicis-lycopersici* in rice and tomato (Additional file 1: Figure S1) and significantly promoted the growth of *A. thaliana* (Additional file 2: Figure S2). To further determine whether the PGPR HN09-induced systemic resistance was dependent on the metabolites of this organism, we examined the ability of HN09-C (containing only HN09 cells), HN09-S (containing only HN09 metabolites) and HN09-FB (containing both HN09 cells and metabolites) to induce resistance against DC3000. The leaves of plants treated with HN09-C showed typical symptoms of bacterial speck disease, and the yellow or water-soaked spots are surrounded by chlorosis (Fig. 1a). HN09-FB and HN09-S significantly reduced pathogen growth in leaves (Fig. 1b). These data suggested that HN09 metabolites indeed induced systemic resistance against DC3000 in plants, but HN09 bacterial cells did not have the same effect.

### Compound spectroscopic data and structure determination

**Fig. 2:**
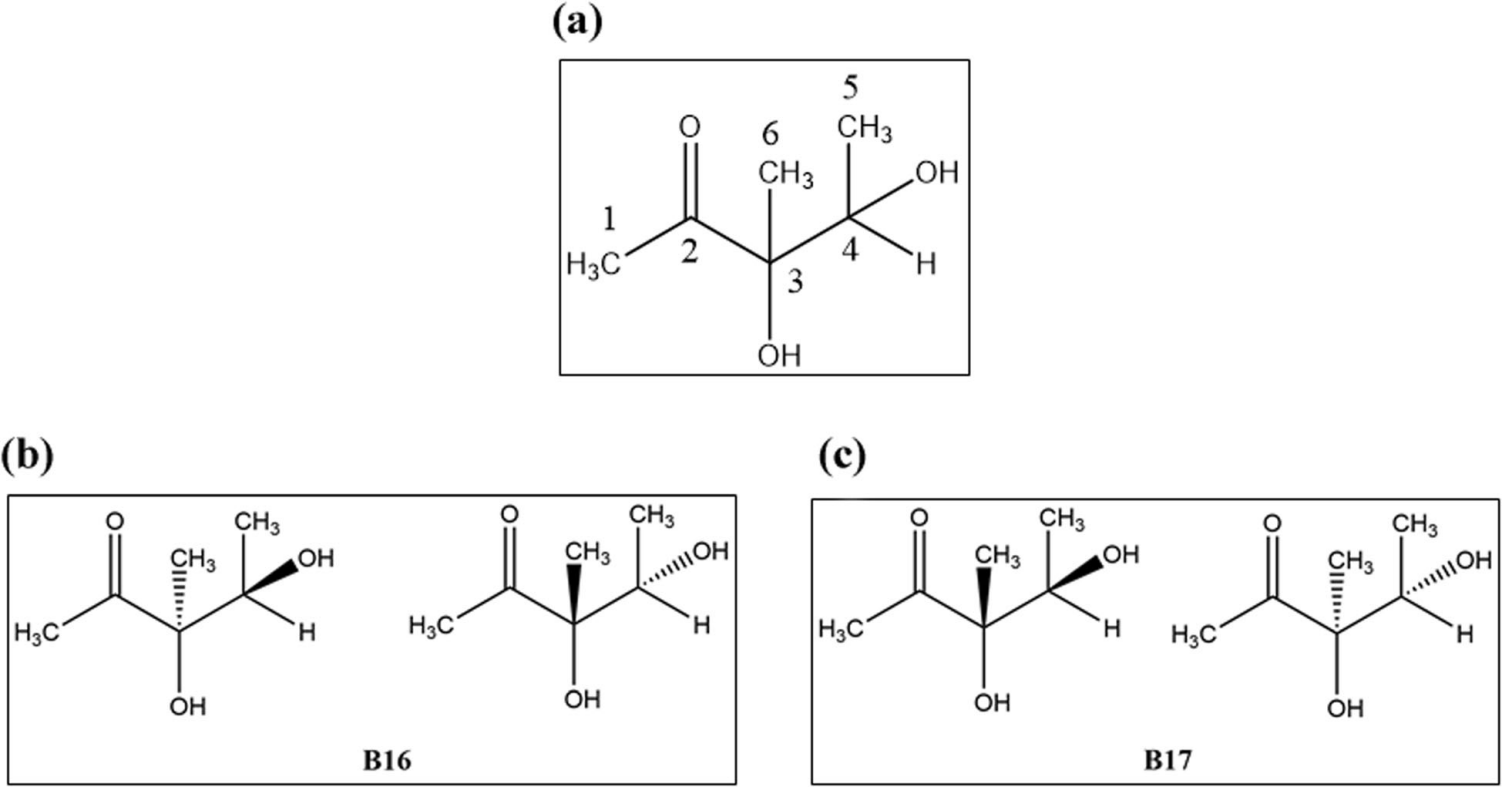
(**a**) The structure of 3,4-dihydroxy-3-methyl-2-pentanone. (**b**) B16 was a 1:1 mixture of (3R,4S)- and (3S,4R)-3,4-dihydroxy-3- methyl-2-pentanone. (**c**) B17 was a 1:1 mixture of (3S,4S)- and (3R,4R)-3,4-dihydroxy-3- methyl-2-pentanone

To identify active compounds among HN09 metabolites, we separated the metabolites from the HN09 culture filtrate and obtained two active compounds, namely, B16 and B17. B16 was obtained as a colourless oil ;${\left[a\right]}_{\mathrm{D}}^{24}$ ±0° (c = 0.202, MeOH); positive EI-MS m/z:133 [M + H]+, 155 [M + Na] + 1; 1H NMR (600 MHz, MeOD) δ 3.83 (q, J = 6.0 Hz, 1H, H^− 4^), 2.24 (s, 3H, H-1), 1.28 (s, 3H, H-6), 1.10 (d, J = 6.0 Hz, 3H, H-5)。13C NMR (150 MHz, MeOD) δ 215.81(C-2), 82.77(C-3), 72.61(C-4), 26.57(C-1), 21.39(C-6), 17.78(C-5) (Fig. 2).

B17 was also obtained as a colourless oil ;${\left[a\right]}_{\mathrm{D}}^{24}$ ±0° (c = 0.277, MeOH); positive EI-MS m/z: [M + H] + =133, [M + Na] + =155; 1H NMR (600 MHz, MeOD) δ 3.97 (q, J = 6.0 Hz, 1H, H-4), 2.26 (s, 3H, H-1), 1.21 (s, 3H, H-6), 1.18 (d, J = 6.0 Hz, 3H, H-5). 13C NMR (150 MHz, MeOD) δ 215.23(C-2), 82.94(C-3), 72.50(C-4), 25.81(C-1), 22.02(C-6), 17.05(C-5) (Fig. 2).

The NMR data for B16 and B17 were very similar to those for 3,4-dihydroxy-3-methyl-2-pentanone [14]. Based on their optical rotation values, it was clear that both compounds were racemates composed of enantiomeric isomers. Therefore, B16 was identified as a 1:1 mixture of (3R,4S)- and (3S,4R)-3,4-dihydroxy-3-methyl-2-pentanone, and B17 was identified as a 1:1 mixture of (3S,4S)- and (3R,4R)-3,4-dihydroxy-3-methyl-2-pentanone (Fig. 2) [14].

### B16 and B17 induced resistance to *P. syringae* DC3000 in *A. thaliana*

**Fig. 3:**
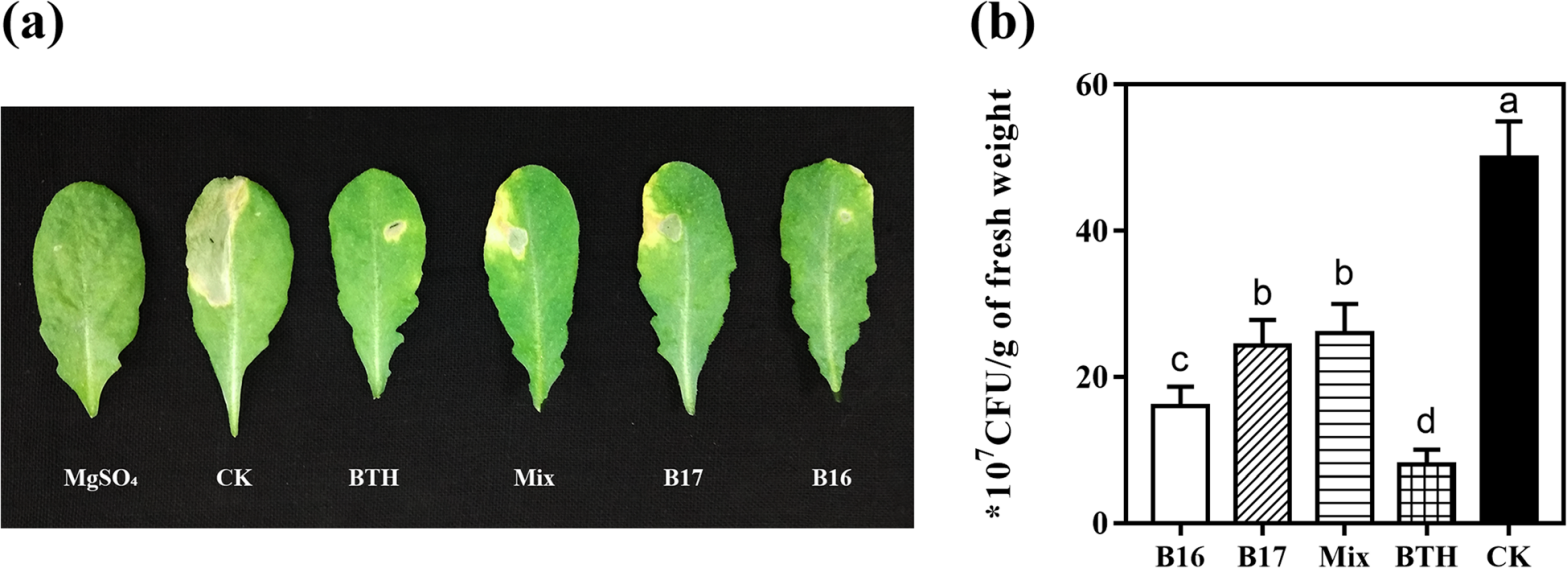
B16 and B17 induced resistance to *P. syringae* DC3000 in *A. thaliana.* Four-week-old *A. thaliana* plants were treated with B16, B17, Mix, 100 μΜ BTH, and 10 mM MgSO_4_. After 24 h, leaves were injected with a cell suspension of DC3000 at OD_600_ = 0.001 (**a**) Representative plants of the B16, B17, Mix, BTH, MgSO_4_ and water treatments were photographed 5 days post inoculation (dpi), with BTH as a positive control, and MgSO_4_ and water as a negative control. (**b**) Colony density of DC3000 in the leaves of *A. thaliana* plants from different treatments. Values are the average CFU per gram of leaf, each treatment had 9 plants, and 3 leaves per plant were obtained for quantification of DC3000 density. Different letters indicate statistically significant differences between treatments (least significant difference test; *P* < 0.05)

Whether B16, B17 and their 1:1 mixture (Mix) can trigger ISR to DC3000 in *A. thaliana* plants were tested. Water was taken as the negative control, and ISR chemical benzothiadiazole (BTH) was the positive control. Five days after inoculation with DC3000, the plants treated with mock had typical DC3000 infection symptoms. The leaves were yellowing or had water-soaked spots which were surrounded by extensive chlorosis (Fig. 3a). Compared with the mock-treated plants, the disease area of the plants treated with B16- and B17 was much smaller. Moreover, in the plants treated with B16, B17, and Mix, the density of DC300 in leaves decreased by 67.03, 52.18 and 48.6% 5 days after DC3000 inoculation compared with that in the mock-treated plants (Fig. 3b). In the BTH-treated plants, the density of DC3000 decreased by 83.43% (Fig. 3b). Interestingly, B16 exhibited significantly higher biocontrol activity against DC3000 than B17 and the mixture treatment.

To determine whether B16 and B17 had direct antibacterial activity against pathogens, we monitored the growth of DC3000 in liquid medium containing 100 μM B16, B17, Mix, or the commercial fungicide Thiram (Table 1). Only B16 weakly reduced DC3000 growth, while Thiram completely eliminated the growth of the bacterium. This result excluded the antimicrobial activity of B16 and B17.

**Table 1:** Antibacterial activity of different elicitors against DC3000 in vitro

Treatment	OD (±SD)	Inhibition (%)
B16	0.721 ± 0.153	11.19
B17	0.988 ± 0.208	−21.71
Mix	0.967 ± 0.285	−19.13
Thiram	0.141 ± 0.018	82.57
CK	0.811 ± 0.157	–

### B16 and B17 induced callose deposition in *A. thaliana*

**Fig. 4:**
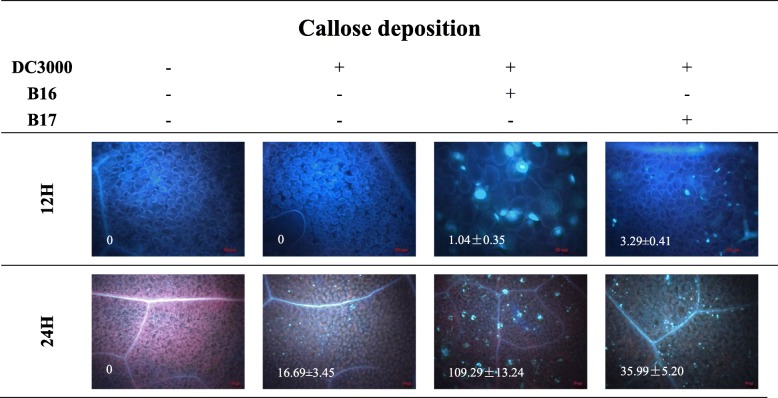
B16- and B17-induced callose deposition in *A. thaliana***.** B16 and B17 prime the activation of callose deposition in the leaves of *A. thaliana* Col-0 plants upon DC3000 attack. *A. thaliana* Col-0 plants were inoculated with DC3000 at 5 dpt, and the leaves were sampled at 24 hpi. Callose deposition was observed under light and epifluorescence microscopes. Each treatment had 6 plants, and all experiments were performed three times, with similar results obtained

The priming mechanism of B16 and B17 for the enhanced activation of cellular defense responses in *A. thaliana* plants was examined. SA- or BTH- pretreated plants producing more callose deposits around the infection site [15, 16]. After inoculation with DC3000 for 12 h, callose deposition in the B16- and B17-treated *A. thaliana* leaves was observed, and the areas were 1.04 and 3.29 mm^2^, respectively; however, callose deposition was observed in neither the CK treatment nor the DC3000-inoculated leaves at the same time point (Fig. 4). At 24 hpi, these defence responses were not observed in the CK treatment, but the B16- and B17-treated plants exhibited larger callose deposition areas (109.29 and 35.99 mm^2^, respectively) than the plants inoculated with DC3000 alone (16.69 mm^2^) (Fig. 4). Importantly, B16 induced stronger callose deposition than B17.

### Defence-related enzyme activity

**Fig. 5:**
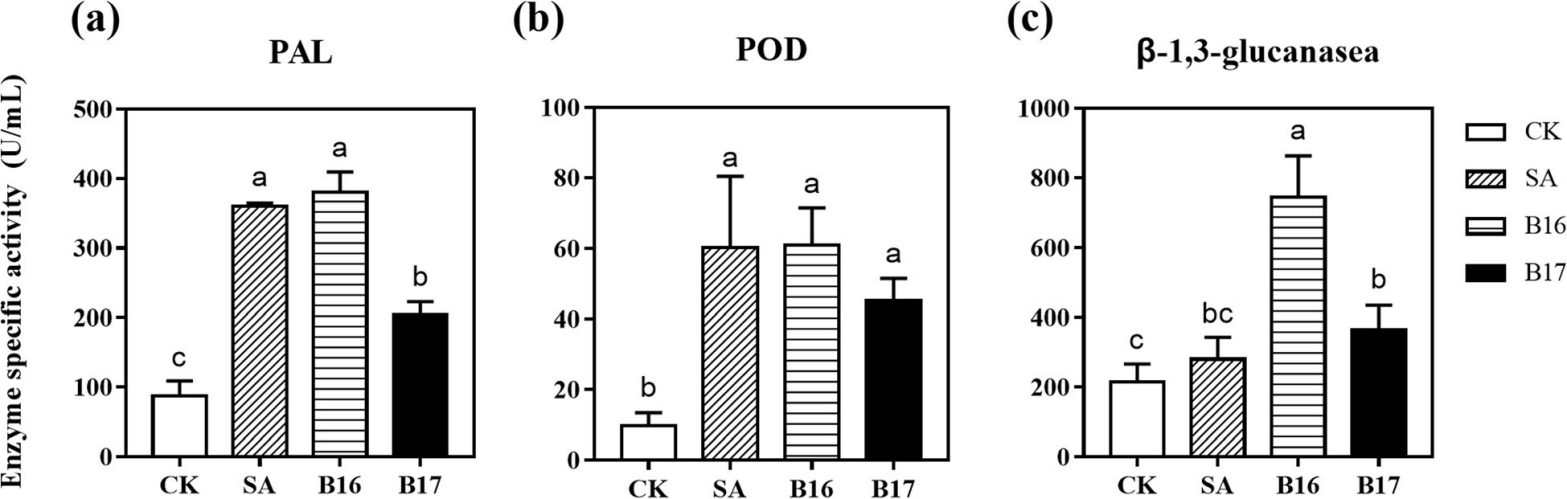
Defence-related enzyme activity. Specific activities of (**a**) PAL, (**b**) POD and (**c**) β-1,3-glucanase after 100 μΜ B16 and B17 treatment in Col-0 *A. thaliana* plants. Each treatment had 12 plants, and all experiments were performed three times, with similar results obtained. Different letters indicate statistically significant differences between treatments (least significant difference test; *P* < 0.05)

To further verify the induction of disease resistance by B16 and B17, the activity of key defence-related enzymes in plants that were treated with B16 and B17 for 24 h was analysed. As a vital enzyme in phenolic biosynthesis, phenylalanine ammonia-lyase (PAL) connects primary metabolism (shikimic acid pathway) and secondary metabolism (phenylpropanoid pathway), catalyses the first step in phenylpropanoid biosynthesis and plays an essential role in defence responses [17]_._ The activity of PAL was significantly higher in the B16- and B17-treated plants than in the water-treated control, and the B16-treated plants showed higher activity than the B17-treated plants, and the activity was equal to that of the SA-treated positive control (Fig. 5a). BTH is a derivative of SA, and both of SA and BTH could be set up as positive control for this experiment. It would be better if used BTH as a positive control to be more consistent. But the phenomenon of SA increased the defence-related enzyme activities had been better described than those in BTH. So, we used SA as positive control in this experiment. The early stages of plant resistance response involve two reactive oxygen species (ROS), H_2_O_2_ and O_2_^−^, which can be induced by abiotic or biotic elicitors, but excess ROS are deleterious to plant cells [18]. Oxidative stress can be inhibited by peroxidase (POD). With higher ROS level, H_2_O_2_ is decomposed by POD by the oxidation of co-substrates like phenolic compounds. The construction, rigidification and eventual lignification of cell walls involve POD enzyme, which can avoid the damage of plant tissues [19]. In our study, in the B16- and B17-treated plants, the POD enzyme activity was significantly higher than that in the water-treated control and was equal that in the SA-treated positive control, which demonstrated that B16 and B17 could successfully elicit plant defence responses, similar to the phytohormone SA (Fig. 5b). β-1,3-glucan and other components of fungal cell walls may be hydrolysed by pathogenesis-related proteins like β-1,3-Glucanases, thus damaging propagule or pathogenic structures. Generally speaking, the accumulation of β-1,3-glucanase can indicate the induced resistance. Our results indicated that the activities of β-1,3-glucanases in *A. thaliana* plants were markedly enhanced by B16 and B17, which indicated that the B16 and B17 treatments could increase plant resistance (Fig. 5c).

### B16 and B17 induced defence-related gene expression in *A. thaliana*

**Fig. 6:**
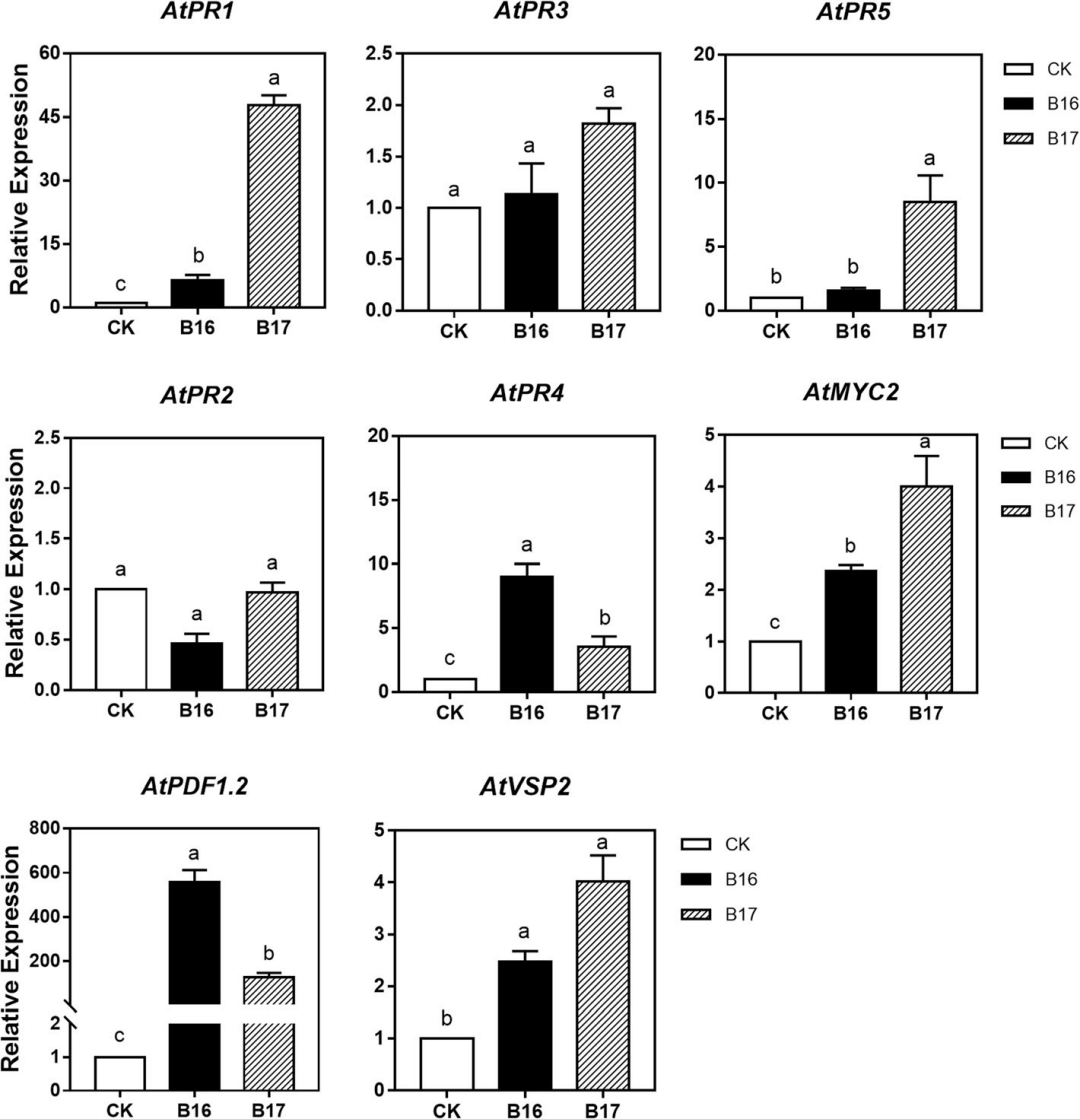
B16- and B17-induced defence-related gene expression in *A. thaliana*. Four-week-old plants were treated with B16 and B17, and leaf samples were harvested at 24 h to extract total RNA. Each treatment had 12 plants and 3 biological replicates, and every biological replicate contained 4 plants. The results of qRT-PCR analysis of defence-related gene transcript levels in response to B16 and B17. The values are the means ± standard deviations. Different letters above the bars indicate significant differences (*P* < 0.05)

The induction patterns of marker genes for these pathways in plants exposed to B16 and B17 were further studied, so that the role of SA-, JA- and ET-related signal transduction pathways in systemic resistance induced by B16 and B17 can be defined clearly. B16 and B17 were used to treat the plants for 24 h. The transcription of JA-inducible genes *AtVSP2* and *MYC2,* JA/ET-inducible genes *PR3* and *PDF1.2,* ET-inducible gene *PR4,* and SA-inducible genes *PR1*, *PR2*, and *PR5* was analysed by real-time quantitative RT-PCR (Fig. 6). The plants treated with B16 and B17 had higher expression of the SA-dependent marker gene *PR-1* (more than 6-fold and 47-fold, respectively). B17 treatment also increased the expression of *PR5*(8.5-fold). In the plants treated with B16 and B17, the expression of ET-inducible marker gene *PR4*, JA-inducible genes *MYC2* and JA/ET-inducible marker gene *PDF1.2* was upregulated. In the experiments, JA/ET-inducible gene *PR3* and SA-inducible gene *PR2* were not expressed noticeably (< 2-fold).

Notably, although B16 and B17 simultaneously induced the upregulation of SA- or JA/ET-dependent genes, the expression levels were different obviously. The JA/ET-dependent marker gene *PDF1.2* was expressed at 557.05 and 125.51 times higher levels in the B16- and B17-treated plants, respectively, than in the control. However, the SA-dependent marker gene *PR1* was expressed at 6.48- and 47.72- times higher level in the B16- and B17-treated plants, respectively, than in the control. This result indicated that JA and ET might be involved in the B16- and B17-induced resistance, though with different patterns at the molecular level.

### B16- and B17-triggered transcriptomic changes

To elucidate the molecular mechanism of B16- and B17-induced plant disease resistance and differences in biological activity, we profiled the responses triggered in 14-d-old plate-grown *A. thaliana* seedlings treated with 100 μM B16 or B17 for 12 and 24 h by mRNA sequencing (mRNA-seq). As controls, we used a mock treatment (solvent only). MeJA (which is readily converted to JA) was set up as a positive control because the JA-dependent gene *PDF1.2* was induced to the highest level among all the tested genes in B16- and B17-treated Col-0 *A. thaliana*. All the differentially expressed genes (DEGs) are listed in Additional file 4: Data set S1.

**Fig. 7:**
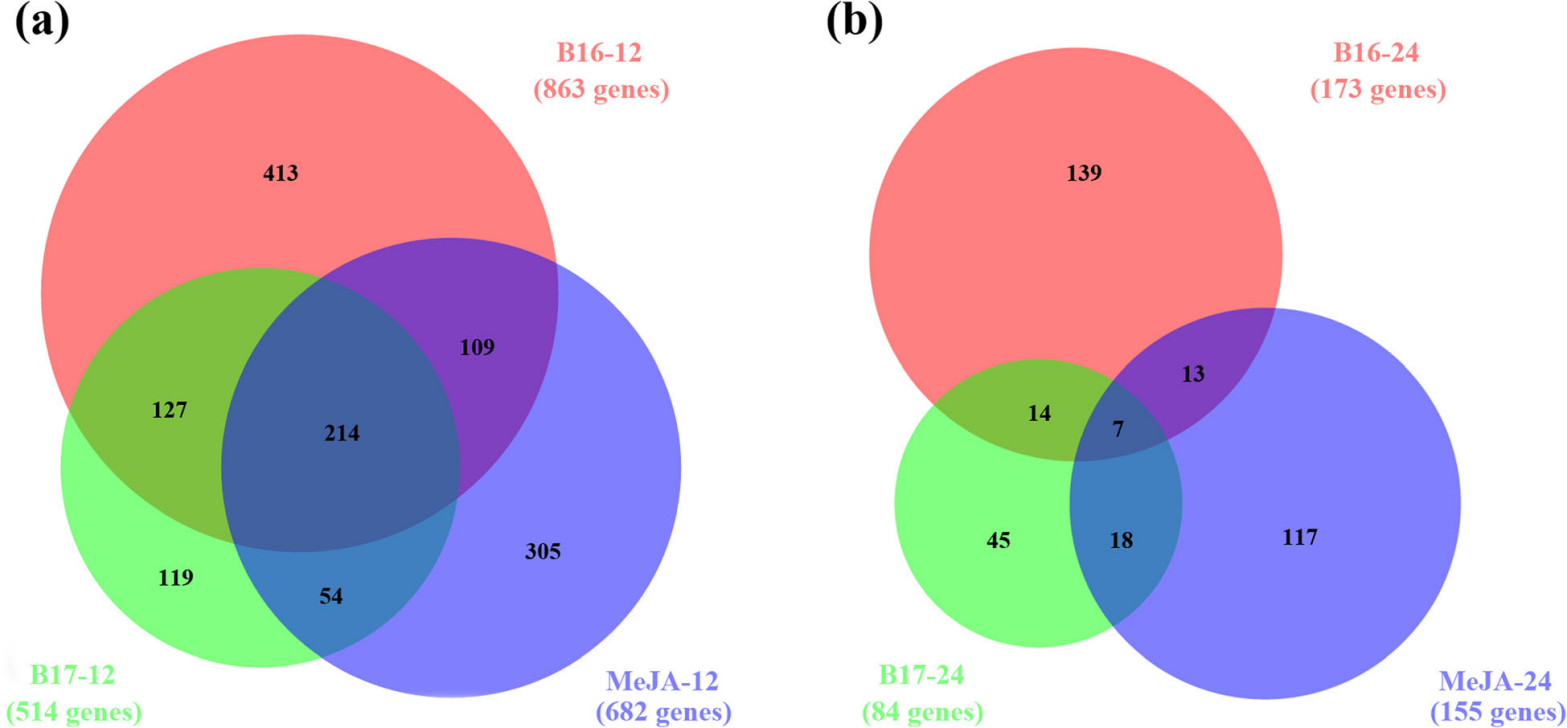
B16- and B17-triggered transcriptomic changes. Venn diagram analysis highlights the differences and similarities between the gene sets that were triggered by 100 μM B16 and B17 in *A. thaliana* Col-0 plants. (**a**) Gene sets from plants treated for 12 h with different compounds. (**b**) Gene sets from plants treated for 24 h with different compounds

At 12 h, a total of 863 genes exhibited significantly altered transcript levels in the B16 treatment (B16–12), with 162 genes upregulated and 702 genes downregulated. In the B17–12 (treated with B17 for 12 h) treatment, the number of differentially expressed genes (DEGs) was slightly lower (514 DEGs) than that in the B16–12 treatment, with 98 upregulated genes and 416 down-regulated genes. As a control, the exogenous MeJA-12 treatment (treated with MeJA 12 h) induced 682 DEGs, including 323 DEGs (more than 47%) that were also induced in the B16 treatment and 268 DEGs (more than 39%) that were also induced in the B17 treatment (Fig. 7), showing a large overlap among B16, B17 and MeJA. This finding suggests that B16 and B17 might mimic some JA functions to activate the transduction of plant defence signals.

Highly significantly enriched Gene Ontology (GO) terms in the B16–12 and B17–12 set determined based on the Gene Ontology database suggested that the collective roles of the B16–12 genes were mainly enriched in “regulation of plant-type hypersensitive response (GO:0010363)”, “response to abscisic acid (GO:0009737)”, “protein targeting to membrane (GO:0006612)”, “response to ethylene (GO:0009723)” and “response to jasmonic acid (GO:0009753)”. In addition to “response to ethylene (GO:0009723)”, “response to jasmonic acid (GO:0009753)” and “regulation of plant-type hypersensitive response (GO:0010363)”, B17–12 genes were enriched in the GO term “response to chitin (GO:0010200)” (Table 2). Chitin, which is a polymer of N-acetyl-D-glucosamine (NAG), is a common PAMP causing plant immunity [20, 21]. B17-induced DEGs significantly enriched the GO terms “response to chitin” and “protein targeting to membrane”, suggesting that B17 may be identified by immune receptor proteins on the cell membrane as a PAMP and could activate the chitin signal transduction pathway. The GO term “response to chitin” was enriched in only the B17-treated plants and not in the B16-treated plants, indicating that the 3,4-dihydroxy-3-methyl-2-pentanone receptor had structural specificity, which may explain why B16 and B17 showed significant differences in the above experiment. B16- and B17-induced DEGs mostly enriched defence-related GO terms, such as those associated with the regulation of plant-type hypersensitive responses or responses to phytohormones, once again showing that B16 and B17 are successful elicitors in plants.

**Table 2:** Set of *A. thaliana* genes significantly differentially expressed in response to B16 or B17 treatment in plate-grown Col-0 seedlings

Treatment	Enriched GO terms^a^	NumDEInCat	NumInCat	*P* value
B16–12	Protein targeting to membrane (GO:0006612)	40	364	3.55E-11
	Regulation of plant-type hypersensitive response (GO:0010363)	40	366	3.55E-11
	Response to abscisic acid (GO:0009737)	43	450	3.12E-10
	Response to ethylene (GO:0009723)	30	253	3.49E-09
	Response to jasmonic acid (GO:0009753)	26	262	2.35E-06
	Response to oxidative stress (GO:0006979)	21	181	3.76E-06
	Systemic acquired resistance (GO:0009627)	23	243	2.83E-05
B17–12	Response to ethylene (GO:0009723)	30	253	2.72E-14
	Regulation of plant-type hypersensitive response (GO:0010363)	33	366	7.93E-13
	Systemic acquired resistance (GO:0009627)	27	243	1.54E-12
	Defense response to fungus (GO:0050832)	30	314	1.75E-12
	Response to jasmonic acid (GO:0009753)	26	262	3.76E-11
	Salicylic acid biosynthetic process (GO:0009697)	23	208	7.49E-11
	Systemic acquired resistance, salicylic acid mediated signalling pathway (GO:0009862)	25	251	7.49E-11
	Response to chitin (GO:0010200)	31	420	3.19E-10
	Jasmonic acid mediated signalling pathway (GO:0009867)	25	275	4.91E-10
	Defence response to bacterium (GO:0042742)	28	359	9.09E-10
B16–24	Generation of precursor metabolites and energy (GO:0006091)	15	67	2.73E-17
	DNA-templated transcription, elongation (GO:0006354)	15	124	2.38E-13
	Photosynthesis (GO:0015979)	17	189	2.38E-13
	Translation (GO:0006412)	11	364	0.002746
B17–24	Generation of precursor metabolites and energy (GO:0006091)	7	67	1.14E-06
	Photosynthesis (GO:0015979)	9	189	1.96E-06
	DNA-templated transcription, elongation (GO:0006354)	7	124	2.92E-05
	Systemic acquired resistance, salicylic acid mediated signalling pathway (GO:0009862)	8	251	0.000184
	Regulation of hydrogen peroxide metabolic process (GO:0010310)	7	183	0.000250
	Response to bacterium (GO:0009617)	6	172	0.002197

^a^Listed are the biological functions of the significantly enriched GO terms based on the Gene Ontology database (http://www.geneontology.org/)

At 24 h, the sets of B16–24 and B17–24 DEGs were substantially smaller than at previous timepoints (173 genes and 84 genes, respectively, Table 2). As a control, MeJA-induced DEGs also exhibited reduced numbers at 24 h (only 155 genes). When stimulated by biotic or abiotic stress, plants quickly transmit the defence signal throughout their bodies by adjusting the Ca^2+^ concentration, thereby inducing a defence response, such as increased expression of defence marker genes and accumulation of JA and JA-Ile [22]. This process is rapid and takes only 30 to 60 min to complete [22]. Therefore, at 24 h, we observed that the number of B16-, B17- and MeJA-induced DEGs decreased. However, the DEGs induced by B16 and B17 featured the enriched term “DNA-templated transcription, elongation (GO:0006354)” (Table 2), which indicates that the early defence signals activate the transcription and translation of downstream defence genes. The DEGs were also enriched in the term “generation of precursor metabolites and energy (GO:0006091)” and the terms related to photosynthesis. There is correlation between defense responses and increasing demands for energy. In the defense responses of plants, plant respiration is highly stimulated [23]. According to the studies on plant defence and photosynthesis, photosynthetic metabolism is locally inhibited in plant defense responses [24, 25]. Photosynthetic apparatus has high sensitivity to ROIs produced during the defence-associated oxidative burs [24].

Taken together, the results show that there were two clearly recognizable trends for 3,4-dihydroxy-3-methyl-2-pentanone-induced transcriptional changes at both 12 and 24 h: (1) B16 and B17 triggered a substantial number of significantly differentially expressed genes and (2) enriched defence-related GO terms, such as regulation of plant-type hypersensitivity, response to oxidative stress and response to phytohormones (Table 2). In addition, B16, B17 and MeJA co-induced abundant DEGs, suggesting that B16 and B17 act as partial agonists of the plant defence hormone JA.

## Discussion

After sensing characteristic microbe- or pathogen-associated molecular patterns (MAMPs or PAMPs), plant cell surface pattern recognition receptors will activate the pattern-triggered immunity. PGPR-produced metabolites can enhance plant self-immunity [26]. It was found that 2,3-Butandiol isolated from *Bacillus subtilis* GB03 trigger ISR, and transgenic lines of *B. subtilis* that secreted reduced levels of 2,3-butanediol conferred decreased the resistance to *A. thaliana* against pathogen infection [1], which indicates that PGPR metabolites contribute substantially to ISR. *Bacillus subtilis* HN09, as a rhizobacterium that can promote plant growth, was isolated from neem rhizospheric soil. In our previous work, we found that HN09 could successfully control tomato *Fusarium* crown and root rot [27], but the mechanism underlying the induction of disease resistance is not clear. Kutschera et al. [28] reported that immunity in *A. thaliana* plants can be triggered by bacterial medium-chain 3-hydroxy fatty acid metabolites. Hence, we tested whether HN09 metabolites could induce plant resistance. The data showed that only treatment with HN09-C did not enhance *A. thaliana* resistance to DC3000, but the HN09-FB and HN09-S treatments significantly reduced DC3000 growth in leaves (Fig. 1), suggesting that HN09-induced disease resistance depends on its metabolites.

For the separation of HN09 secondary metabolites, we obtained two compounds, namely, B16 and B17 (Fig. 2), which could induce systemic resistance in *A. thaliana*. B16 has the same chemical structure as B17, but these compounds differ in stereoconfiguration. Numerous studies have shown that differences in stereoconfiguration can significantly affect the affinity between a compound and its receptor protein, which in turn affects the bioactivity of the compound [29, 30]. This phenomenon has also been observed in the study of elicitors. It was observed that the (*2R,3R*) form plays a main role in 2,3-butanediol-induced resistance to *Erwinia carotovora* subsp. *carotovora*, but the (*2S,3S*) form does not play such a role [1, 6]. Kutschera et al. [28] also found that (R)-3-hydroxydecanoic acid induced stronger immune signalling than (S) -3-hydroxydecanoic acid. In the *A. thaliana* phenotype experiment, we observed similar results, demonstrating that the 4-dihydroxy-3-methyl-2-pentanone threo-isomer B16 exhibited more effective biocontrol of DC3000 than the erythro-isomer B17 (Fig. 3). In addition, B16 was also better than Mix, which indicates that one of the B16 (3*R,4S*) or (3*S,4R*) isomers may be the optimal configuration for triggering plant ISR against DC3000.

In the callose deposition experiment, B16- and B17-treated plants advanced and enhanced cellular defence responses against the pathogen DC3000 (Fig. 4). Callose deposition is an important cellular defence response in plants, preventing the spread of pathogens [2]. *Pseudomonas syringae* effectors can induce callose deposition in susceptible *A. thaliana* plants [31]. Moreover, we observed a significant difference between B16 and B17 at the cellular level in the triggering of plant ISR (Fig. 4), which was accordance with the phenotypic experiment results.

Generally, ISR depends on the JA or ET signalling pathway, while systemic acquired resistance (SAR) is dependent on the SA signalling pathway [22]. However, many new studies have demonstrated that ISR also involves SA signalling pathway. For instance, SA signalling pathway plays an important role in inducing systemic resistance to colonization by *Trichoderma asperellum* SKT-1. JA/ET and SA signalling pathways act together for induction of resistance against the culture filtrate of this organism [32]. Similarly, the expression of SA, JA and ET signalling marker genes was primed by ISR triggered by a long-chain volatile isolated from *Paenibacillus polymyxa* E681 [33]. In our study, the SA, JA and ET signalling pathway gene transcription levels were upregulated in the B16- and B17- treated plants (Fig. 6), which indicate that the B16- and B17-induced defence in *A. thaliana* involves both JA/ET and SA signalling. On the other hand, RNA-Seq data also exhibited that among B16- and B17-induced DEGs the GO terms related to response to JA or ET (GO:0009723; GO:0009753, Table 2 and Additional file 5: Data set S2) were significantly enriched. Also, few DEGs enriched SA-related GO terms (GO:0009862; GO:0009697 Additional file 5: Data set S2). The JA response is a typical mechanism of ISR induced by beneficial microorganisms. In addition, B16 and B17 were isolated from the PGPR *Bacillus subtilis* HN09. Thus, we hypothesize that the JA/ET signalling pathway plays a dominant role in B16- and B17-elicited plant immunity and that the SA signalling pathway is also involved in this process. However, SA-JA-ET signalling is associated with complex cross-talk, and we did not have enough evidence to elucidate the specific mechanism of action between B16/B17 and the SA-JA-ET signalling pathway in this study, but we look forward to further investigating this mechanism in subsequent research.

Interestingly, B16 and B17 are a pair of chiral isomeric compounds that not only differ in configuration but also differ significantly in effect. With a high degree of structural specificity in both receptors and complexes, the stereoconfiguration of a compound greatly affects its biological activity. The natural product (+)-coronatine induces protein-protein interactions between CORONATINE INSENSITIVE1 (COI1; the F-box subunit of the skp/Cullin/F-box-type ubiquitin ligase complex) and JASMONATE ZIM DOMAIN (JAZ) transcriptional repressor proteins, causing plant growth inhibition or senescence and plant defence responses [29]. However, a stereochemical isomer of coronatine can interact with only JAZ9 and JAZ10, not other JAZ isoforms, without a tradeoff between growth and defence [34]. In this study, the differences in the binding mode and intensity of B16 or B17 and receptors upstream of the plant immune system might have caused the differences in downstream defence responses, such as callose deposition, defence-related enzyme activity and defence-related gene expression.

RNA-seq showed that many DEGs were induced after B16 or B17 treatment compared with the water-treated control (Fig. 7). Through GO enrichment analysis, we found that these DEGs are significantly enriched in pathways associated with plant disease resistance (Table 2), once again showing that B16 and B17 are successful elicitors in plants.

## Conclusion

We isolated and identified a new elicitor, 3,4-dihydroxy-3-methyl-2-pentanone, from the metabolites of the plant growth-promoting rhizobacterium *Bacillus subtilis* HN09. 3,4-dihydroxy-3-methyl-2-pentanone induced plant resistance and exhibits conformational specificity, and the threo-isomer B16 and erythro-isomer B17 induced significant differences in disease resistance at the phenotypic, cellular and molecular levels. Our data showed that B16 induced a stronger defence response than B17 in all the experiments and that the plant hormone SA, JA and ET signalling pathways were involved in this defence response. This study provided new insights into the roles of bacterial metabolites as initiators of defence responses in plants, further opening up the possibility of identifying novel chemical inducers through rational design.

## Methods

### Plants, bacterial strains, and growth conditions

In the experiments, *A. thaliana* (ecotype Columbia, Col-0) seeds were acquired from the Arabidopsis Biological Resource Center (https://abrc.osu.edu/). After soaked overnight in cold water at 4 °C, the seeds were planted in peat soil in pots. Two-week-old seeds were grown in 200-mL pots under fluorescent lights (100 μ E m^− 2^ s^− 1^, 23 °C, 10 h of light/14 h of dark).

We grew the challenge pathogen *Pseudomonas syringae* pv. *tomato* DC3000 overnight at 28 °C in liquid King’s medium B (KB) [2] (20 g•L^− 1^ peptone, 1.5 g•L^− 1^ MgSO_4_, 1.5 g•L^− 1^ K_2_HPO_4_, and 10 mL•L^− 1^ glycerinum) which contained 50 mg•L^− 1^ rifampicin. Centrifugation (2000×g, 3 min) was conducted to pellet the cultured DC3000 cells, which were washed with sterile water and resuspended in 10 mM MgSO_4_ containing the surfactant Silwet L-77 (Sigma) at 0.01% (v/v). Afterwards, it was adjusted to OD_600_ = 0.01 for gradient dilution to the required concentration.

In this experiment, *Bacillus subtilis* HN09 was tested, which was isolated from neem rhizospheric soil from the Insecticidal Plants Arboretum of South China Agricultural University, Guangdong Province, China [27]. We grew HN09 in yeast extract-peptone-dextrose (YPD) liquid medium (3 g•L^− 1^ beef extract, 5 g•L^− 1^ yeast powder, 20 g•L^− 1^ peptone,12.5 g•L^− 1^ glucose) for 24 h at 28 °C. The bacterial cells were pelleted by centrifugation (2000×g, 3 min), resuspended in sterile 0.85% NaCl, and adjusted to OD_600_ = 0.10 for the HN09 cell treatment (HN09-C). The remaining supernatant containing HN09 metabolites was filter sterilized with a 0.2 μm syringe-driven filter as the NH09 supernatant treatment (HN09-S). The HN09 fermentation (HN09-FB) treatment contained both HN09 bacterial cells and metabolites, and the concentration was adjusted to OD_600_ = 0.10.

### Metabolite extraction and isolation

petroleum ether, CHCl_3_, EtOAc, and *n*-BuOH were used to extract the culture filtrate of HN09 sequentially three times to generate dried petroleum ether-soluble (29.87 g), CHCl_3_-soluble (16.03 g), EtOAc-soluble (30.6 g), and n-butyl alcohol-soluble (80.15 g) extracts. The EtOAc-soluble extract was subjected to passage over a silica gel column (100–200 mesh) which was eluted with CHCl_3_-methanol mixtures of increasing polarities (100:0–90:10–80:20–70:30–60:40–50:50, v/v). A total of 10 fractions (E1-E10) were obtained. Fraction E4 (7.4 g), which was obtained by elution with CHCl_3_–MeOH (80:20), was rechromatographed on a Develosil ODS (10 μm, Nomura Chemical Co. Ltd., Japan) column. Moreover, 16 subfractions (E4–1–E4–16) was obtained by eluting Fraction E4 with MeOH–H_2_O mixtures (3:7, 1:1, and 7:3). preparative HPLC was used to separate the subfraction E4–1, which was run with a Shimadzu RID-10A refractive index detector and a Shimadzu LC-6 AD pump by taking a YMC-pack ODS-A C18 column (5 μm, 250 × 20 mm) and MeOH-water mixture (7:93, v/v) as the mobile phase to generate B16 (2.86 g) and B17 (1.76 g).

### Plant treatment and pathogen inoculation

After *A. thaliana* growth in pots for 2 weeks, 10 mL of HN09-S, HN09-C and HN09-FB was poured over the surface of each pot. An equal volume of sterile 10 mM MgSO_4_ was used for control treatment. Five days after treatment, the leaves were sprayed with a cell suspension of the virulent pathogen *Pseudomonas syringae* pv. *tomato* DC3000 at OD_600_ = 0.01 until fine droplets cover all leaves to challenge-inoculate the plants of all treatments. For the HN09 metabolite B16 and B17 treatments, four-week-old *A. thaliana* plants were treated with 100 μM B16, B17, Mix (a mixture of B16 and B17), BTH and MgSO_4_ by spraying the leaves. Twenty-four hours after the treatment, the DC3000 pathogen was inoculated by injecting bacteria (diluted to OD_600_ = 0.001) into leaves through the stomata on the undersides of the leaves. After inoculation, the plants were maintained for 3 days at 100% relative humidity in a dew chamber and then transferred to a growth chamber (100 μ E m^− 2^ s^− 1^, 23 °C, 10 h of light/14 h of dark).

### Quantification of DC3000 in *A. thaliana* leaves

The leaves were sampled on the fifth day after inoculation (dpi) to determine the density of DC3000 in *A. thaliana* leaves. Each treatment had 12 plants, and 3 representative leaves per plant were obtained for quantification of DC3000 density. All samples were weighed, soaked for 30 s in 75% ethanol for surface sterilization, washed with sterile distilled water three times, and then homogenized with a sterile mortar and pestle with 10 mM MgSO_4_. Appropriate dilutions were plated onto KB medium supplemented with 100 mg•L^− 1^ cycloheximide and 50 mg•L^− 1^ rifampicin, which were incubated at 28 °C. After 48-h incubation, we counted the number of colonies of the rifampicin-resistant pathogen strain DC3000. The bacterial density was expressed as CFU per gram of leaf fresh weight (CFU•g^− 1^). This experiment was conducted four times. A least significant difference (LSD) test (*P* = 0.05) was carried out for mean comparison. LSD results and standard errors were recorded.

### Callose deposition

*A. thaliana* leaves were sampled at 24 hpi and treated as described by Niu [2], so that callose deposition can be determined. Each treatment had 6 plants, and 3 representative leaves per plant were obtained for analysis of callose deposition. Briefly, all leaves were soaked in 40 mL of destaining solution (ethanol/water/lactic acid/glycerol/phenol = 8:1:1:1:1 (v/v/v/v/v)) and vacuum-infiltrated for 10 min. Then, the chlorophyll was cleared by incubating the samples for 30 min in a 60 °C water bath. After rinsing with water, the chlorophyll-free leaves were soaked for 2 to 4 h in the dark in 0.01% (w/v) aniline blue staining solution with 150 mM K_2_HPO_4_ (pH 9.5). Finally, leaves were observed and photographed under an Olympus BX51 microscope with a UV excitation filter (Olympus, Shinjuku, Tokyo, Japan). The pixels of the callose deposition area were calculated by Adobe Photoshop 2015 CC and then converted to area according to the number of pixels per square millimetre.

### Enzyme activity assay

After ground in liquid nitrogen, *A. thaliana* leaves (0.5 g) were homogenized with 1.5 mL of phosphate buffer (50 mM, pH 7.8). We centrifuged the extracts at 10,000 rpm for 15 min at 4 °C. The activity of phenylalanine ammonia-lyase (PAL) and peroxidase (POD) was determined by supernatant. POD enzyme activity was assayed as follows [35]. The assay mixture contained 0.1 mL of enzyme solution, 0.2 mL of 4% (v/v) guaiacol, 0.2 mL of 0.46% (v/v) H_2_O_2_, and 2.5 mL of 0.1 M sodium phosphate buffer (pH 7.0). We recorded the increase in absorbance at 470 nm at 25 °C for 3 min. One unit of POD activity was the amount of enzyme leading to a change of 0.01 in absorbance per minute and calculated according to the following formula: POD enzyme activity [U/(g ⋅ min)] = ΔOD_470_ ⋅ D/(0.01 ⋅ W ⋅ t) (ΔOD_470_: change in absorbance; D: dilution factor; W: fresh weight; t: reaction time). We incubated the assay mixture containing 0.1 mL of enzyme extract, L-phenylalanine (2 mM) and sodium borate buffer (20 mM, pH 8.8) at 30 °C for 1 h for PAL activity. The 0.01-unit increase in absorbance at 290 nm caused by the formation of trans-cinnamate was measured. PAL enzyme activity [U/(g ⋅ h)] = ΔOD_290_ ⋅ D/(0.01 ⋅ W ⋅ t) [36]. β-1,3-A previously described method was modified to determine glucanase activity [37]. 5 mL of sodium acetate buffer (50 mM, pH 5.0) was used to homogenize one gram of leaf sample, which was further centrifuged at 4 °C for 20 min at 8000 rpm to obtain the crude extract. In order to detect β-1,3-glucanase, the mixture of 100 mL of crude extract and 100 mL of 1% laminarin was incubated for 60 min at 37 °C. The sample was heated for 10 min in boiling water to terminate the reaction. The amount of enzyme required to catalyse the production of 1 mg of glucose equivalent h^− 1^ at 37 °C was one unit of β-1,3-glucanase activity.

### Real-time RT-PCR analysis

We performed quantitative RT-PCR as previously described [27] in order to monitor defence-related gene expression. The Plant RNA Kit (OMEGA, USA) was used to extract total RNA from the cotyledons. The ratio of the absorbance at 260 and 280 nm was determined by ultraviolet spectrophotometry to measure RNA quality. In order to remove contaminating genomic DNA, the first-strand complementary DNA (cDNA) was synthesize by 1 μg of total RNA digested by DNase I (NEB, USA) through iScript reverse transcription (Bio-Rad, USA). Primers were synthesized by BGI Tech Solutions Co., Ltd. (Shenzhen, Guangdong, China). Additional file 3: Table S1 lists the primer sequences [5]. We used a CFX Connect real-time PCR detection system (Bio-Rad, USA) to perform PCR in optical 96-well plates with a total volume of 20 μL, containing 10 μL of 2× IQ SYBR Green Supermix reagent (Bio-Rad, USA), 0.5 μL of each of the two gene-specific primers (10 pmol μL^− 1^),1 μL of cDNA, and 8 μL ddH_2_O in a final volume of 20 μL. The ΔΔCT (cycle threshold) method was employed to normalize the relative expression levels of the selected genes to those of the reference gene *AtUBQ5* using the following equation: amount of target = 2^−ΔΔCT^, where ΔΔCT = (C_T,target_ – C_T,ubq5_)_Treatment_ – (C_T,target_ – C_T,ubq5_)_Ck_ [38]. Each treatment had 12 plants and 3 biological replicates, and every biological replicate contained 4 plants.

### RNA-Seq analysis

The rosette leaves were dipped into a mock, B16, B17, or MeJA (Sigma, Shanghai, China) solution to treat the four-week-old *A. thaliana* plants. There were 0.015% (v/v) Silwet L77 and 100 μM B16, B17 and MeJA in the B16, B17 and MeJA solutions, respectively. The mock solution contained 0.015% (v/v) Silwet L77 (Sigma). After treatment for 12 h and 24 h, we harvested the sixth leaf (counted from the oldest true leaf to the youngest leaf) from individual *A. thaliana* plants, which was snap frozen in liquid nitrogen [39]. Leaves from three potted plants for each treatment and each time point were mixed as a single sample for sequencing. RNA-seq was completed by the Hengchuang Gene Technology Company (ShenZhen, Guangdong, China). The raw RNA-Seq read data are deposited in the Sequence Read Archive (http://www.ncbi.nlm.nih.gov/sra/; BioProject ID: PRJNA555596). The NEBNext® Ultra™ RNA Library Prep Kit for Illumina® (NEB, USA) was used to generate sequencing libraries according to the manufacturer’s instructions. DEGSeq was used for differential expression analysis [40]. *q* value (or FDR) < 0.01 and |log2 (foldchange)| > 1 were the thresholds for significant differential expression. GOseq was used for Gene Ontology (GO) enrichment analysis of differentially expressed genes (DEGs), and the bias of gene length was corrected. GO functional analysis provides GO functional enrichment analysis for DEGs and GO functional classification annotation for DEGs. The biological functions of enriched GO terms were determined based on the Gene Ontology database (http://www.geneontology.org/).

## Additional files

Additional file 1: **Figure S1.** *Bacillus subtilis* HN09 induces defence responses in multiple plant species against diverse pathogens. (**a**) HN09 enhances systemic resistance to *Fusarium oxysporum* f. sp. *radicis-lycopersici* in tomato. Tomato plant roots drenched with HN09 (optical density at 600 nm OD_600_ = 0.1) display a reduction in disease severity in leaves relative to CK-treated (solvent only) plants 7 dpi (*n* = 4). Red boxes mark the symptoms developing from the base of the plant. (**b**) HN09 enhances resistance to *Magnaporthe oryzae* GD00–193 in rice. The roots of two-week-old rice of the generally susceptible variety Lijiangxintuanheigu (LTH) and resistant variety HNBL9 were treated with HN09 at OD_600_ = 0.1; seven days later, the leaves were sprayed with a cell suspension of GD00–193 at 1 × 10^5^ CFU/mL. Quantitative real-time RT-PCR was used to quantitatively analyse GD00–193 growth on leaves. χ^2^ tests were performed for difference significance analysis; ** indicates significant differences at *P* < 0.01; *** *P* < 0.001. The data shown are representative of at least three independent experiments. (TIF 386 kb)

Additional file 2: **Figure S2.** Growth promotion of *A. thaliana* Col-0 by HN09. One-week-old *A. thaliana* Col-0 sterile plants were treated with HN09, DH5α or sterile water, with the latter two serving as controls. Each treatment had 30 plants. (**a**) Representative plants of each treatment were photographed 14 days post treatment. (**b**) Dry weight of each treatment plant. The data presented were from a representative experiment that was repeated three times with similar results. Different letters indicate statistically significant differences between treatments (Fisher’s least significant difference; *P* < 0.05). (TIF 8405 kb)

Additional file 3: **Table S1.** Primers used in this study. (DOCX 15 kb)

Additional file 4: **Dataset S1.** List of DEGs from the B16, B17 and JA treatments in this study. (XLSX 67 kb)

Additional file 5: **Dataset S2.** Enriched GO terms from the B16, B17 and JA treatment sets of DEGs. (XLSX 29 kb)

## Data Availability

The data sets supporting the results of this article are included within the article and its additional files. The biological functions of enriched GO terms were determined based on the Gene Ontology database (http://www.geneontology.org/). The raw RNA-Seq read data are deposited in the Sequence Read Archive (http://www.ncbi.nlm.nih.gov/sra/; BioProject ID: PRJNA555596).

## Abbreviations

BTH: Benzothiadiazole
DEGs: Differentially expressed genes
ET: Ethylene
GO: Gene ontology
ISR: Induced systemic resistance
JA: Jasmonic acid
MAMPs: Microbe-associated molecular patterns
MeJA: Methyl jasmonic acid
PAL: Phenylalanine ammonia-lyase
PAMPs: Pathogen-associated molecular patterns
PGPR: Plant growth-promoting rhizobacterium
POD: Peroxidase
ROS: Reactive oxygen species
SA: Salicylic acid
SAR: Systemic acquired resistance
