# Supplementary figures and images for: The stereoisomeric Bacillus subtilis HN09 metabolite 3,4-dihydroxy-3-methyl-2-pentanone induces disease resistance in Arabidopsis via different signalling pathways
Source: BMC Plant Biol. 2019 Sep 5;19:384. doi: 10.1186/s12870-019-1985-6 (PMC6727425; doi:10.1186/s12870-019-1985-6)

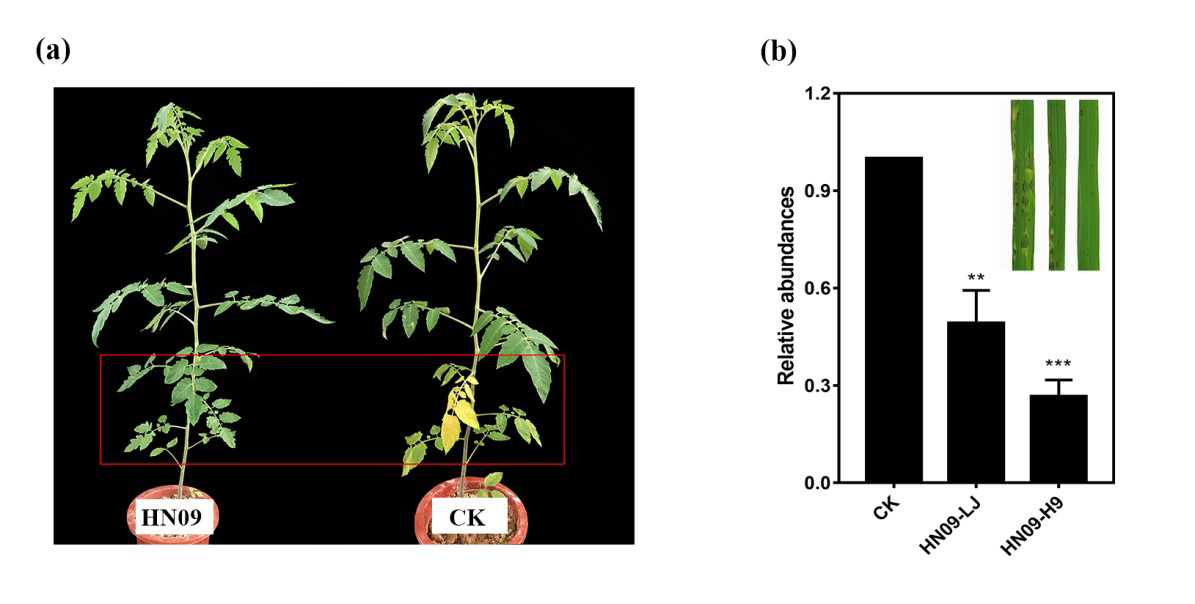

Supplement: Supplementary file 1 — Figure S1. Bacillus subtilis HN09 induces defence responses in multiple plant species against diverse pathogens. (a) HN09 enhances systemic resistance to Fusarium oxysporum f. sp. radicis-lycopersici in tomato. Tomato plant roots drenched with HN09 (optical density at 600 nm OD600 = 0.1) display a reduction in disease severity in leaves relative to CK-treated (solvent only) plants 7 dpi (n = 4). Red boxes mark the symptoms developing from the base of the plant. (b) HN09 enhances resistance to Magnaporthe oryzae GD00–193 in rice. The roots of two-week-old rice of the generally susceptible variety Lijiangxintuanheigu (LTH) and resistant variety HNBL9 were treated with HN09 at OD600 = 0.1; seven days later, the leaves were sprayed with a cell suspension of GD00–193 at 1 × 10^5^ CFU/mL. Quantitative real-time RT-PCR was used to quantitatively analyse GD00–193 growth on leaves. χ2 tests were performed for difference significance analysis; ** indicates significant differences at P < 0.01; *** P < 0.001. The data shown are representative of at least three independent experiments. (TIF 386 kb) [file 12870_2019_1985_MOESM1_ESM.tif]

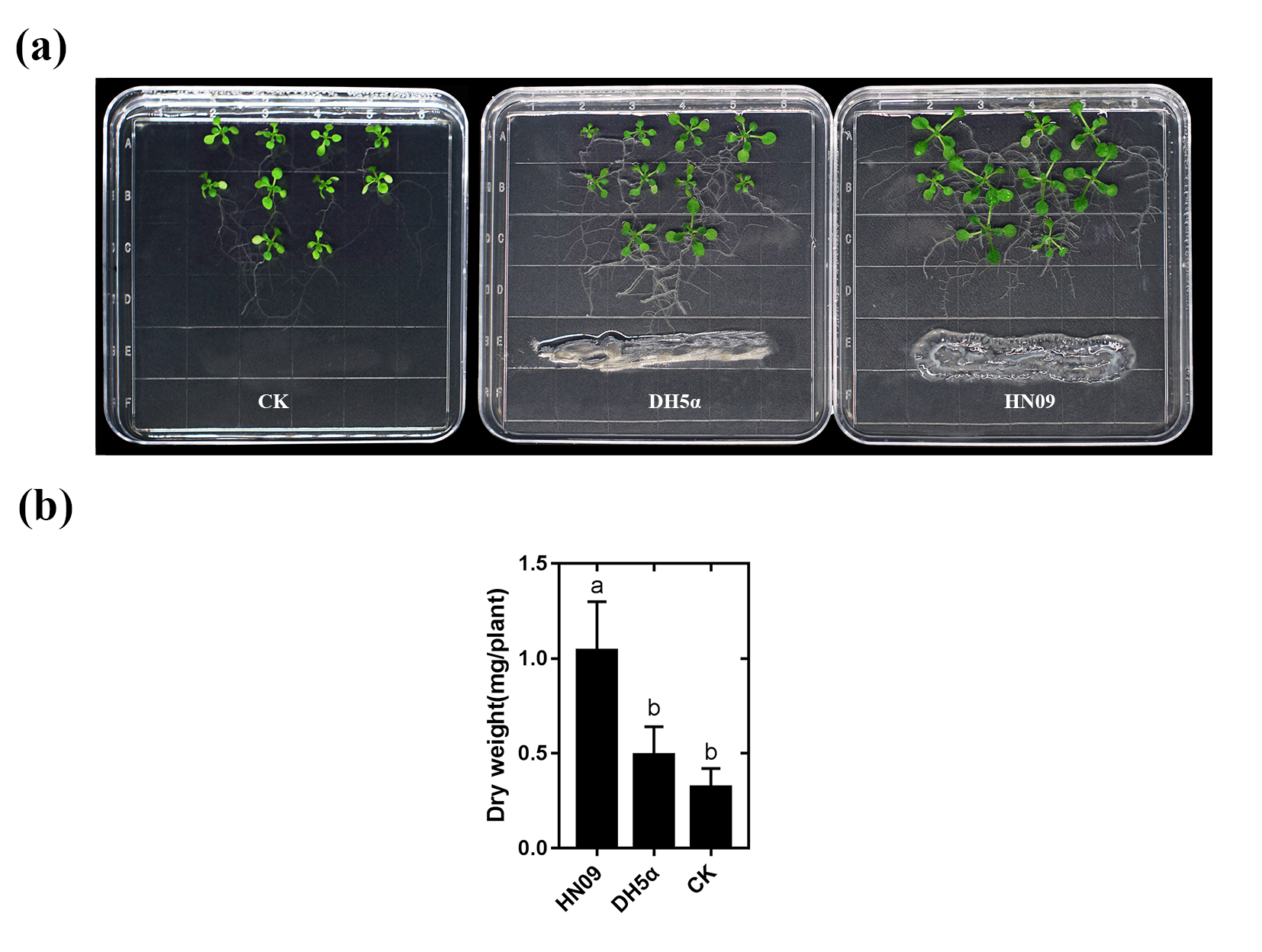

Supplement: Supplementary file 2 — Figure S2. Growth promotion of A. thaliana Col-0 by HN09. One-week-old A. thaliana Col-0 sterile plants were treated with HN09, DH5α or sterile water, with the latter two serving as controls. Each treatment had 30 plants. (a) Representative plants of each treatment were photographed 14 days post treatment. (b) Dry weight of each treatment plant. The data presented were from a representative experiment that was repeated three times with similar results. Different letters indicate statistically significant differences between treatments (Fisher’s least significant difference; P < 0.05). (TIF 8405 kb) [file 12870_2019_1985_MOESM2_ESM.tif]
