# Supplementary material for: The stereoisomeric Bacillus subtilis HN09 metabolite 3,4-dihydroxy-3-methyl-2-pentanone induces disease resistance in Arabidopsis via different signalling pathways
Source: BMC Plant Biol. 2019 Sep 5;19:384. doi: 10.1186/s12870-019-1985-6 (PMC6727425; doi:10.1186/s12870-019-1985-6)
Supplement: Supplementary file 3 — Table S1. Primers used in this study. (DOCX 15 kb) [file 12870_2019_1985_MOESM3_ESM.docx]

**Additional file 3: Table S1. Primers used in this study.**

| **Gene name** | **Gene ID** | **Primer sequence (5´→3´)^a^** | **Product size (bp)** |
| --- | --- | --- | --- |
| ***PR-1*** | At2g14610 | F:TGGTCACTACACTCAAGTTGTT  R:GCTTCTCGTTCACATAATTCCC | 128 |
| ***PR-2*** | At3g57260 | F:CGTTGTGGCTCTTTACAAACAA  R:AGCTCTGAACGTTTTCTTGAAC | 188 |
| ***PR-5*** | At1g75040 | F:AGGATTTGAATTGACTCCAGGT  R:CCATCGCCTACTAGAGTGAATT | 201 |
| ***PR-3*** | At3g12500 | F:GGTAACACCGAACCATACTGTA  R:TTGAAATGATGCCCGAAAGATC | 103 |
| ***PR-4*** | At3g04720 | F:ATAATCCGGCGCAGAATAATTG  R:CAGTTACTGCAGCATTTGTTCT | 195 |
| ***PDF1.2*** | At5g44420 | F:CTTATCTTCGCTGCTCTTGTTC  R:TGGGAAGACATAGTTGCATGAT | 183 |
| ***AtVSP2*** | At5g24770 | F:CAAAGGACTTGCCCTAAAGAAC  R:GTCTTCTCTGTTCCGTATCCAT | 114 |
| ***UBQ5*** | At3g62250 | F:GAAGATCCAAGACAAGGAAGGA  R:CTTCTTCCTCTTCTTAGCACCA | 163 |

**^a^F:** forward; **R:** reverse.
